# Innovative Molecular Targets and Therapeutic Approaches in Nonalcoholic Fatty Liver Disease/Nonalcoholic Steatohepatitis (NAFLD/NASH) 3.0

**DOI:** 10.3390/ijms25074010

**Published:** 2024-04-03

**Authors:** Mariapia Vairetti, Giuseppe Colucci, Andrea Ferrigno

**Affiliations:** 1 Department of Internal Medicine and Therapeutics, University of Pavia, 27100 Pavia, Italy; andrea.ferrigno@unipv.it; 2 Division of Gastroenterology and Hepatology, Department of Pathophysiology and Transplantation, Fondazione IRCCS Ca’ Granda Ospedale Maggiore Policlinico, University of Milan, 20122 Milan, Italy; giuseppe.colucci@guest.unimi.it

The aim of this Special Issue is to provide an update on the diagnosis and treatment of nonalcoholic fatty liver disease (NAFLD), which is the most prevalent liver disease worldwide; however, there are still no specific treatment agents. 

As numerous advancements have been made to clarify the causes and pathogenesis of this disease, a more practical and clinical-based approach has been proposed, consisting of the adoption of “positive criteria” to diagnose the disease. This has resulted in the renaming of NAFLD as MAFLD (Metabolic-Associated Fatty Liver Disease) [1].

Over the past three decades, the prevalence of NAFLD has increased from 25% to 38% [2]. Although NAFLD was initially identified as a “Western” disease, a recent meta-analysis conducted by Younossi et al. showed that the highest prevalence was found in Latin America with 44.37% (30.66%–59.00%), followed by the Middle East and North Africa (MENA) (36.53%, 28.63%–45.22%), South Asia (33.83%, 22.91%–46.79%), and South-East Asia (33.07%, 18.99%–51.03%). North America (31.20%, 25.86%–37.08%), East Asia (29.71%, 25.96%–33.76%), Asia Pacific 28.02% (24.69%–31.60%), and Western Europe 25.10% (20.55%–30.28%) showed a lower prevalence [2]. The increasing prevalence of NAFLD in many developing countries may be linked to the excessive consumption of sugary foods, especially those containing high-fructose corn syrup found in sugar-sweetened beverages. This habit has been associated with postprandial hypertriglyceridemia and visceral adiposity contributing to insulin resistance and NAFLD [3,4]. These results suggest that a significant number of patients with chronic disease may progress into steatohepatitis (NASH) or fibrosis, with the risk of developing cirrhosis and hepatocellular carcinoma (HCC).

This Special Issue highlights that, although the pathogenesis of NAFLD has been extensively studied, the mechanisms associated with the progression of the disease to NASH have yet to be fully elucidated. NAFLD comprises a spectrum of chronic liver diseases varying from isolated hepatic triglyceride accumulation and steatosis to lipotoxicity and mitochondrial dysfunction, causing progressive liver damage and fibrosis [5].

Of note, the liver tissues of NAFLD patients showed ultrastructural mitochondrial lesions, mitochondrial DNA damage, and reduced ATP production [6]. Moreover, the increase in mitochondrial reactive oxygen species (ROS) induces oxidative damage to mitochondrial proteins and DNA, decreases mitochondrial membrane potential, produces inflammation, and promotes programmed cell death/apoptosis [7]. These events are responsible for the reduction in liver function. Recently, markers of hepatic mitochondrial biogenesis, autophagy, fission, and fusion were found to be significantly decreased in patients with NAFLD/NASH [6]. Moreover, connections between the liver and other organs, including the adipose tissue and the gut, have been found, contributing to the multifactorial nature of NAFLD [5].

An ideal therapy for the treatment of NAFLD would be aimed toward a reduction in steatosis hepatic inflammation and the prevention of fibrosis, while advising patients on the need to adopt appropriate dietary and lifestyle changes and monitor their metabolic parameters [8]. Rodent models were used to study the complexity of the factors involved in NAFLD development and progression. Experimental models have provided crucial insights into the characterization of NAFLD pathogenesis and have served to screen and test new therapeutic approaches. Human-like NAFLD features have been induced in preclinical models such as genetic models, i.e., the leptin-deficient mouse model (ob/ob) or leptin receptor-deficient mice (db/db) [8]. Using a specific diet alongside the genetic model, these animals reproduce the histological and metabolic modifications of human NASH [8].

To date, no drug capable of reversing NAFLD and preventing its evolution towards NASH has been registered. However, numerous molecules are currently being studied in phase II and III clinical trials, and some agents have shown potential efficacy in reducing steatosis, inflammation, and fibrosis. Current pharmacological agents for the treatment of NAFLD/NASH targeting de novo lipogenesis, β-oxidation, and bile acid metabolism have been reported by Parlati et al. [8]. These include Obeticholic acid, Cilofexor, Tropifexor, and Elafibranor that act as antagonists for nuclear receptors or Firsocostat, TVB-2640, Aramchol, and Pradigastat, inhibitors of acetyl-CoA carboxylase (Acc), fatty acid synthase (Fas), stearoyl-CoA desaturase (Scd)-1, and diacylglycerol acyltransferase (Dgat)-2, respectively. Other therapies target apoptosis (Emricasan, Selonsertib, and Simtuzumab) or fibrosis (Cenicriviroc and Belapectin (GR-MD-02). Collagen deposition and fibrosis can be prevented by limiting the activation of Kupffer cells and hepatic stellate cells (HSC) [8]. 

The review by Vitulo et al. summarizes different approaches for the treatment of NAFLD with particular attention to the agonists for nuclear factors like peroxisome proliferator-activated receptor (PPAR) and farnesoid X receptor (FXR) and the reverse cholesterol transport system [9]. The review includes a list of PPAR-based drugs as well as FXR-based drugs that are currently being tested in clinical trials. An overview of drugs developed for other conditions, such as incretins, namely GIP and GLP-1, hormones released from the intestine in response to food intake, and thyromimetics, in the case of NAFLD-associated thyroid dysfunction, is also provided. Furthermore, validated natural compounds, such as plant-derived polyphenols, have been considered for their antioxidant and anti-inflammatory properties [9].

In addition to the treatment of NAFLD, its specific diagnosis and monitoring also deserves to be improved. While liver histology remains the gold standard for the diagnosis and staging of NAFLD, new non-invasive methods are needed to better assess and monitor treatment response and disease progression [10]. Several classes of molecules, specific proteins, or metabolites have been proposed as the result of “omics technologies”, based on the analysis of thousands of different molecules [11]. Proteomics and metabolomics have been applied to identify the metabolic profile specific for NAFLD regarding key molecules such as bile acids and glutathione, whose levels are altered during NAFLD onset [12]. Although their limited diagnostic performance has restricted their use, several studies have demonstrated that the combination of several biomarkers could have a clinical application for steatosis diagnosis and staging [11]. 

However, despite these innovations, the pathogenetic mechanisms involved in the progression from NAFLD to NASH is still underexplored. Of note, therapeutic approaches capable of reversing NAFLD and preventing its evolution towards NASH should target either the metabolism, to decrease liver fat deposition, or apoptosis and fibrosis, to limit the progression of NAFLD. Furthermore, although several attempts have been made to identify innovative diagnostic indexes, to date, there are no specific non-invasive biomarkers that are able to accurately diagnose and stage NAFLD across the full spectrum of the disease.
